# *In Situ* Determination of the Effects of Lead and Copper on Cyanobacterial Populations in Microcosms

**DOI:** 10.1371/journal.pone.0006204

**Published:** 2009-07-10

**Authors:** Mireia Burnat, Elia Diestra, Isabel Esteve, Antonio Solé

**Affiliations:** Department of Genetics and Microbiology, Biosciences Faculty, Universitat Autònoma de Barcelona, Barcelona, Spain; University of Sydney, Australia

## Abstract

**Background:**

Biomass has been studied as biomarker to evaluate the effect of heavy metals on microbial communities. Nevertheless, the most important methodological problem when working with natural and artificial microbial mats is the difficulty to evaluate changes produced on microorganism populations that are found in thicknesses of just a few mm depth.

**Methodology/Principal Findings:**

Here, we applied for first time a recently published new method based on confocal laser scanning microscopy and image-program analysis to determine *in situ* the effect of Pb and Cu stress in cyanobacterial populations.

**Conclusions/Significance:**

The results showed that both in the microcosm polluted by Cu and by Pb, a drastic reduction in total biomass for cyanobacterial and *Microcoleus sp*. (the dominant filamentous cyanobacterium in microbial mats) was detected within a week. According to the data presented in this report, this biomass inspection has a main advantage: besides total biomass, diversity, individual biomass of each population and their position can be analysed at microscale level. CLSM-IA could be a good method for analyzing changes in microbial biomass as a response to the addition of heavy metals and also to other kind of pollutants.

## Introduction

Benthic stratified sediments (microbial mats) that cover kilometer long areas of coastal territory are made up of a dense biomass of microorganisms [1]–[5]. The most important microorganism populations living in microbial mats are cyanobacteria, and among them *Microcoleus chthonoplastes* is dominant in marine intertidal microbial mats, hypersaline environments and hot deserts [6]. Cyanobacteria may also be present in environments that are polluted by heavy metals, and have been the object of research into metal biosorption [7], [8] and toxicity studies [9], [10]. However, the role that metals may represent in cyanobacteria populations in microbial mats is unclear due, in part, to the fact that it is difficult in these environments to determine whether the changes produced in the diversity or biomass is the result of the presence of pollutants or to changes in environmental parameters. This problem can be resolved through the use of microcosms (artificial laboratory ecosystems), in which only one variable is changed whilst the others are controlled constantly [11]. Microcosms also are useful for studying the effect of pollutants without involving the risk of liberating these toxic compounds into the natural environment.

Nevertheless, the most important methodological problem when working with microbial mats is the difficulty to evaluate changes produced on cyanobacterial populations that are found in thicknesses of just a few mm depth. One of the main goals of assessing the effects of heavy metals on microbial communities in natural and artificial ecosystems in the laboratory is the search for an effective and reliable bioindicator. Changes in diversity or biomass can usually be considered good indicators for evaluating the effect of metals on bacterial populations. Amongst those most used are molecular techniques and biochemical analysis [12]–[16] but the former do not contribute quantitative data and the latter do not succeed in distinguishing (as is the case with cyanobacteria) distinct morphotypes within the same sample. Moreover, in all cases the methods used involve a considerable manipulation of samples and do not allow differentiation of the individual biomass of microbial populations at micron-scale level.

Optical microscopy techniques such as fluorescence microscopy have also been used [13], [17] but the images obtained using this technique showed high out-of-focus fluorescence. Several years ago, we used CLSM for first time to determine changes in cyanobacterial populations, because this technique avoids the use of long and exhaustive protocols, allows accurate and non-destructive optical sectioning giving results where out-of-focus images were virtually eliminated. Moreover, as cyanobacteria emit natural fluorescence, staining protocols were not required [18]. Nevertheless, this method was manual and required that the limits of every cyanobacterium presented in each summa projection be established manually on screen so as to obtain different morphometric data.

We have recently published a new method based on determining cyanobacterial bimass by the Confocal Laser Scanning Microscopy- Image Analysis (CLSM-IA). This method has made it possible to characterize and identify the photoautotrophic microorganisms and also, with the aid of image-analysis computer systems, to determine their biomass [16]. CLSM-IA allows biomass calculation for microorganisms of a small size, since the limit of the technique's resolution is that generated by a voxel, the smallest unit of a three-dimensional digital image, equivalent to 1.183×10^−3^ mgC/cm^3^ of sediment. This method was especially suitable for the quantitative analysis of a large number of CLSM images generated from benthic sediments in which complex populations of cyanobacteria were abundant, such as unpolluted an oil-polluted microbial mats [19].

In this study, we applied CLSM-IA for first time to cyanobacterial populations living in microcosms in order to determine the effect of heavy metals on these microorganisms, specifically in terms of biomass.

## Results and Discussion

In this paper, we have studied the effect of lead (Pb) and copper (Cu) on the cyanobacteria population, and mainly on *Microcoleus* sp. in microcosms. In order to do so, we have used Confocal Laser Scanning Microscopy (CLSM), which detects the natural fluorescence of photosynthetic pigments, and we have determined the total and differentiated biomass of the cyanobacteria by means of an image analysis program, *Image J* v1.37 (CLSM-IA) [16].

**Figure 1:**
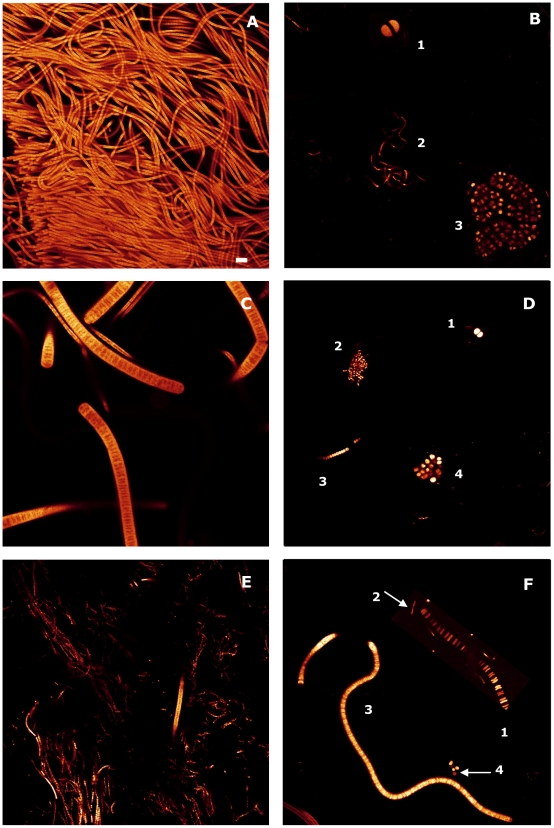
Confocal laser scanning microscopy (CLSM) images of cyanobacteria observed in microcosms. A) *Microcoleus* sp. B1) *Chroococcus*-like B2) *Halomicronema*-like B3) *Myxosarcina*-group C) *Oscillatoria*-like D1) *Synechocystis*-group D2) *Microcystis*-like D3) *Leptolyngbya*-like D4) *Gloeocapsa*-group E) *Halomicronema*-like F1) *Johannesbaptistia-*like F2) *Borzia*-like F3) *Lyngbya*-like F4) *Synechococcus*-group. B, D and F are photographic compositions. Scale bar represents 10 µm.

Firstly, to analyse the diversity of the cyanobacteria, the microcosms were prepared, one of them unpolluted and used as a control experiment, and the other two polluted with 25 mM Pb and 10 mM Cu, respectively. Next, all samples were screened by CLSM. Fig. 1 shows cyanobacteria diversity obtained by this technique. The different types of autofluorescence bacterial cells in the microbial mats samples were classified as different genera of cyanobacteria based on microscopic morphology (Table 1). This table presents the most important characteristics of all the cyanobacteria identified in the present study. *Microcoleus* sp. and *Halomicronema*-like were the most abundant filamentous cyanobacteria. *Microcoleus* sp. presents its trichomes oriented in parallel, enclosed in a common homogenous sheath and the mature end cells are conical. Cells (diameter 3–6 µm) composing the trichomes are longer than they are wide (Fig. 1, A). *Halomicronema*-like has trichomes of a width of around 1 µm wide and does not appear to be constricted at the cross-walls nunder optical microscopy. Cells are always longer than wide (2–8 µm long) and end cells are rounded (Fig. 1, E). The other filamentous cyanobacteria identified were *Oscillatoria*-like (Fig. 1, C), *Lyngbya*-like (Fig. 1, F3), and *Leptolyngbya*-like (Fig. 1, D3) and in less abundance, *Borzia*-like (Fig. 1, F2). The main characteristics of these cyanobacteria are described in Table 1.

**Table 1:** Characteristics of filamentous and unicellular cyanobacteria.

	Microorganisms	Diameter (µm)	Septation	Cell division planes	Gas vaculoes	Sheath or mucilage	Ref.
**Filamentous cyanobacteria**	*Microcoleus chthonoplastes*	3–6	+	binary fission in 1 plane*	−	+	[24]
	*Halomicronema*-like	<1	+	binary fission in 1 plane*	+	+ (thin)	[25]
	*Oscillatoria*-like	4–16	+	binary fission in 1 plane*	−	+	[24]
	*Lyngbya*-like	6–80	+	binary fission in 1 plane*	−	+	[24]
	*Leptolyngbya*-like	<3 (large filaments)	−	binary fission in 1 plane*	−	+	[24]
	*Spirulina*-like	1–5	−	binary fission in 1 plane*	−	−	[24]
	*Borzia*-like	1–2 (short filaments)	+	binary fission in 1 plane*	−	+(thin)	[24]
**Unicellular cyanobacteria**	*Gloeocapsa*-group	3–10	NS**	binary fission in 2 or 3 planes	−	+	[24]
	*Chroococcus*-like	22–32	NS**	binary fission in 2 planes	−	+	[24]
	*Synechocystis*-group	2–6	NS**	binary fission in 2 or 3 planes	−	+	[24]
	*Synechococcus-*group	0.6–2.1	NS**	transverse binary fission in a single plane	−	+	[24]
	*Myxosarcina*-like	3–10	NS**	binary fission in 3 planes	−	+	[24]
	*Microcystis*-like	3–6	NS**	binary fission in 2 (or 3) planes	+	+	[24]
	*Johannesbaptistia*-like	2–3.5	NS**	binary fission in one plane	−	+	[26], [27]

* Binary fission in a single plane at right angles to the long axis (uniserate trichome).

** NS = No Septation.

Unicellular cyanobacteria belonging to the genera *Gloeocapsa*-group (Fig. 1, D4), *Synechocystis*-group (Fig. 1, D1), *Synechococcus*-group (Fig. 1, F4), *Microcystis*-like (Fig. 1, D2) and *Johannesbaptistia*-like were also identified (Table 1). The last microorganism is unicellular and its cells are 3–3.4 µm wide and 2.5–2.8 µm long and arranged in solitary, forming unbranched pseudo-filaments (Fig. 1, F2).

**Figure 2:**
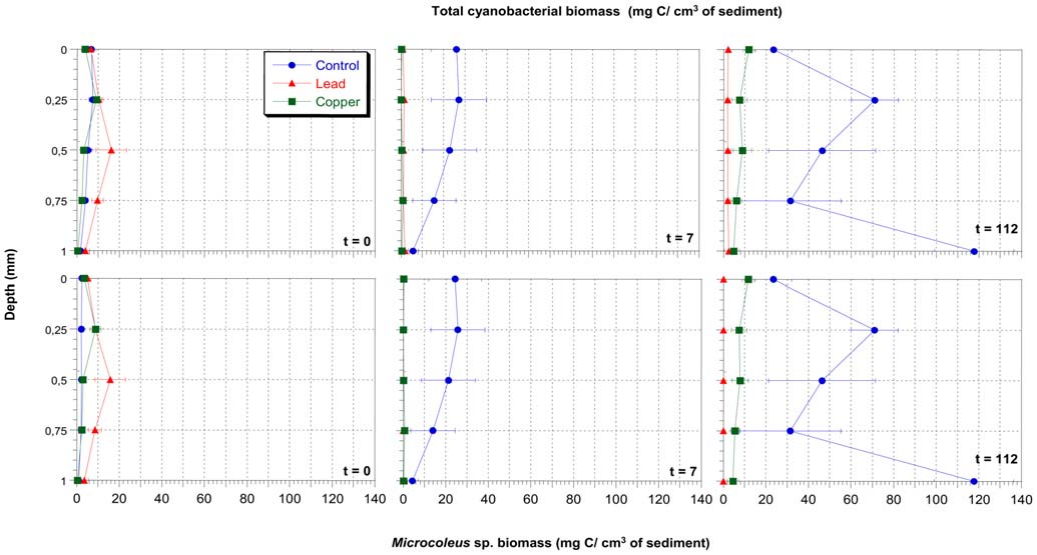
Accurate profiles, at every 250 µm of depth of total cyanobacterial and *Microcoleus* sp. biomass determined by means of CLSM-IA from the control microcosm, and polluted microcosms with 25 mM Pb and 10 mM Cu. *x* axis: depth (mm), *y* axis: time (days).

We applied CLSM-IA to determine total cyanobacterial biomass and *Microcoleus sp*. biomass for evaluating the effects of Pb and Cu in microcosms. The samples from microcosms were taken at specific time and depth intervals (at each 250 µm) from the first mm of depth, as it was in this layer that the greatest cyanobacteria growth was observed (Fig. 2). Four replicates of each depth were done. Biomass and SD variation were calculated from the average of replicates.

In the control microcosm, the profiles of total biomass and those of *Microcoleus* sp. coincided, which showed that this cyanobacterium is dominant, representing in most cases, ≥90% of the cyanobacterial population.

In the microcosm polluted by Pb, a drastic reduction in total biomass from 9.275±4.62 to 1.265±0.38 mgC ·cm^−3^ of sediment was observed 7 days after beginning the experiment. Also, in the microcosm polluted with Pb, a reduction in the *Microcoleus* sp. biomass was determined, from 8.326±4.62 mgC ·cm^−3^ of sediment (day 0) to 0.507±0.25 mgC ·cm^−3^ of sediment (day 10). *Microcoleus* sp. was not detected at t = 112, therefore, the biomass determined at the end of the study was probably mainly due to *Halomicronema*-like. In the microcosm polluted by Cu, the same effect was noticed, a reduction of the total biomass (from 3.813±3.20 to 0.409±0.38 mgC ·cm^−3^ of sediment), and *Microcoleus* sp. biomass (from 3.622±3.14 to 0.199±0.25 mgC ·cm^−3^ of sediment) 7 days after beginning the experiment.

**Figure 3:**
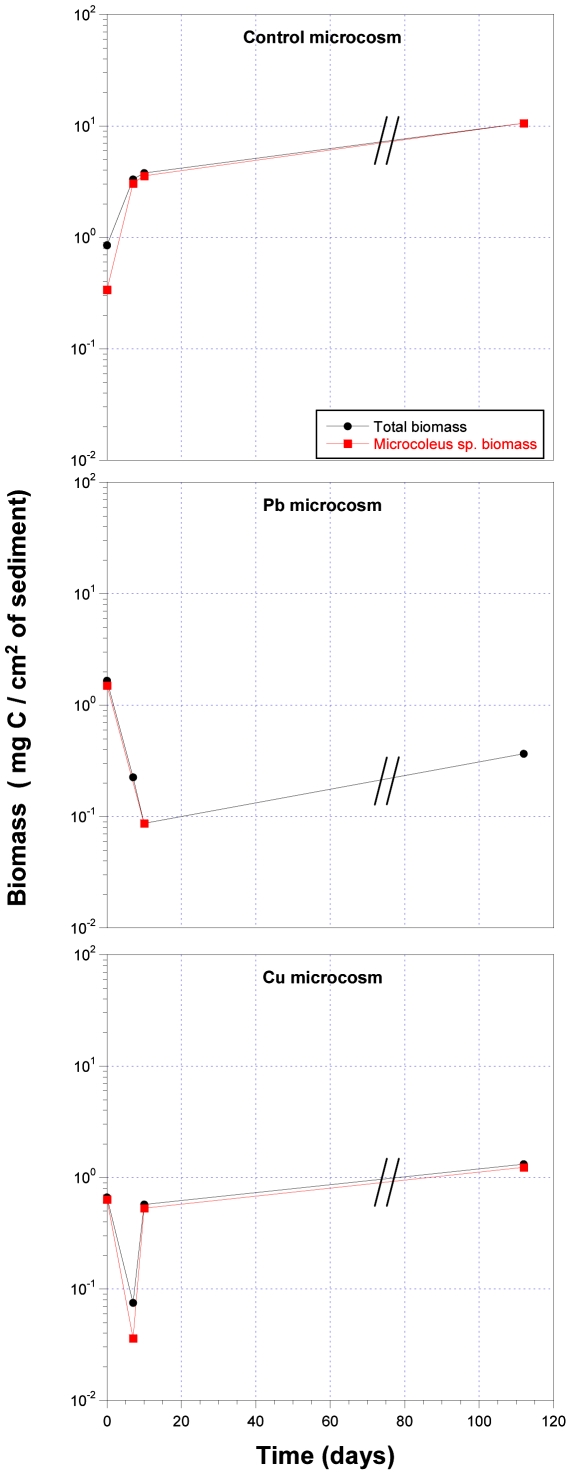
Total cyanobacterial biomass and *Microcoleus* sp. biomass expressed in mg C· cm^−2^ of sediment.

In both cases it is shown that, in the doses of metals used, both Pb and Cu are lethal for cyanobacterial populations and *Microcoleus* sp. after only one week. In Fig. 3, total and *Microcoleus* sp. biomass values are expressed in mg C· cm^−2^ of sediment. This figure shows the lethal effect of Pb and Cu in the above mentioned populations, similar to those represented in Fig. 2. Recently, we have also shown, in laboratory cultures of a *Microcoleus* consortium, a degradation of the photosynthetic pigments, which can be seen through the absence of fluorescence in the emission spectra, and alterations of the cell ultrastructure, such as expanded thylakoids and large intrathylakoidal spaces, in cultures treated with Pb and Cu respectively (M. Burnat, manuscript under review).

Certain reports use changes in community composition and biomass as useful bioindicators for determining the effect of heavy metals on different ecosystems [15], [20], [21], [22]. In the case of biomass, the methods used in these studies encompassed a range of different techniques, such as Phospholipid Fatty Acid patterns (PLFA) [23], Colony-Forming Units (CFU) [14], direct counts with DAPI [13], [22], phospholipid-phosphate analysis (PL-P) [15], and chlorophyll *a* quantification [17], among others. However, not all of these techniques are useful for accurately differentiating the individual biomass (different genera) at micron scales, and moreover, as has been we have already observed, they involve lengthy protocols and sample manipulation.

In conclusion, we believe that CLSM-IA is the best method for estimating cyanobacterial biomass in microbial mats. The method takes advantage of the accurate and nondestructive optical sectioning of confocal microscopy, and autofluorescence of cyanobacteria, presenting a good technique for *in situ* cyanobacterial biomass determination, especially in the application to various benthic sediment samples. According to the data presented in this report, this biomass inspection has two main advantages: first, in addition to total biomass, diversity, individual biomass of each population and their position can be analysed; second, this method can be applied to different kind of natural and artificial ecosystems, including polluted mats. The overall results shown in this paper indicate that Pb and Cu in the concentrations used in this study have a drastic effect on cyanobacterial biomass and mainly on *Microcoleus* sp. in only one week, and that CLSM-IA is a good method for analyzing changes in microbial biomass as a response to the addition of heavy metals and also to other kind of pollutants. Although the method applied would not be suitable to analyse the physiologic state of the cells, we are currently applying the *lambda scan* function of CLSM to analyse the physiological state of the photosynthetic pigments.

## Materials and Methods

### Sampling Site and Microcosms Setup

Samples from microbial mats were obtained from the Ebro delta, located in the northeast of Spain (40° 40'N, 0°40'E), near the Salines de la Trinitat, in the Alfacs Peninsula, on May 2006. Samples were taken in 17 cm×12 cm×8 cm polypropylene boxes and carried to the laboratory. Once in the laboratory, different microcosm experiments were prepared, one of them unpolluted and used as a control experiment, and the other two polluted with 25 mM Pb and 10 mM Cu, respectively. The latter microcosms were maintained in polluted conditions for three days, and afterwards metal solutions were removed. Samples from microcosms were taken with glass cores of diameter of 6 cm on days 0 (before contamination), 7, 10 and 112 for further analysis.

### Preparation of Heavy Metal Stock Solutions

We selected copper and lead on the basis of their biological functions and effects. Copper is an essential metal with known biological functions (micronutrient) in all organisms. In cyanobacteria, copper is a component of plastocyanin in the photosynthetic electron-transport chain; however, at high concentrations, it has toxic effects both on animals and plants. Lead is a toxic metal with no biological function. Lead and copper stock solutions were prepared as Pb(NO_3_)_2_ and CuSO_4_ (Merck KGaA, Darmstadt, Germany) with deionized water and were sterilized by filtration with polycarbonate membrane filters. Heavy metal stock solutions were prepared 24 hours before to be used and kept in darkness at 4°C.

### Confocal Laser Scanning Microscopy (CLSM)

Microcosm core samples for CLSM were fixed in 2.5% glutaraldehyde Sorensen buffer phosphate for 2 h, washed four times with the same buffer phosphate, and kept at 4°C until their analysis. Samples were observed with a 63×1.4 numerical aperture (NA) oil immersion objective lens, excited with a diode 561 nm, and were viewed in a Leica TCS SP2 AOBS (Leica, Heidelberg, Germany). The emission fluorescence was captured between 590 and 800 nm (autofluorescence). Natural fluorescence of chlorophyll *a* and phycobillins was used as a marker to determine cyanobacteria distribution on the three dimensions and to estimate their relative abundance and biomass. Cyanobacteria were identified according to Castenholz [24], Abed et al. [25], Bauer et al. [26] and Kómarek and Haulter [27] based on morphological criteria.

In order to obtain optical series (stacks of images), the *xyz* function of CLSM was used. Images were acquired in 512×512 pixel and 8 bit format. Each stack consisted of 21 optical sections (image *xy*) on the *z* dimension, whose value corresponded to a thickness of 20 µm. The samples from microcosms were taken at depth intervals (at each 250 µm) from the first mm of depth and four replicates of each depth were done. The images obtained were used to determine cyanobacterial biomass in the first mm of depth of the microcosms, where cyanobacteria were dominant.

### Image Analysis and Biomass Estimation

Once the images of cyanobacteria were obtained by CLSM, a free image-processing and analysis program, *ImageJ v1.37*, was used to determine cyanobacterial and *Microcoleus* sp. biomass from mat samples, according to Solé et al. [16]. Stacks of images in their original image format (8-bit and 512×512 pixel) were opened as tiff image sequences and transformed to binary images (black/white), using the threshold adjust commands in which an optimum threshold was required in each of the stacks analysed. Later, a plugin called Voxel Counter created by Wayne Rasband and available on-line, was applied to this software [28]. This specific application calculates the ratio of threshold voxels (cyanobacterial volume) to all voxels (the total sediment volume) from each binary image in every stack. This value (Volume Fraction) was multiplied by a conversion factor of 310 fgC µm^−3^ to convert it to biomass [29], [30].

For *Microcoleus* sp. biomass determination, the binary stacks were obtained using the same optimum threshold as in total biomass determination. In this case, *Microcoleus* sp. was selected from the stack of images, whilst the remaining genera of cyanobacteria were erased. Finally, the *Microcoleus* sp. biomass was determined following the same protocol used for total cyanobacterial biomass estimation.

Total cyanobacterial biomass and the *Microcoleus* sp. biomass profiles were represented using *KaleidaGraphs* software (Synergy Software, USA).
